# Identification of Ureidocoumarin-Based Selective Discoidin Domain Receptor 1 (DDR1) Inhibitors via Drug Repurposing Approach, Biological Evaluation, and In Silico Studies

**DOI:** 10.3390/ph17040427

**Published:** 2024-03-27

**Authors:** Ashraf K. El-Damasy, Hyun Ji Kim, Ahmed A. Al-Karmalawy, Radwan Alnajjar, Mohamed M. Khalifa, Eun-Kyoung Bang, Gyochang Keum

**Affiliations:** 1 Brain Science Institute, Korea Institute of Science and Technology (KIST), Seoul 02792, Republic of Korea; 2 Department of Medicinal Chemistry, Faculty of Pharmacy, Mansoura University, Mansoura 35516, Egypt; 3 Department of Pharmaceutical Chemistry, Faculty of Pharmacy, Horus University-Egypt, New Damietta 34518, Egypt; akarmalawy@horus.edu.eg; 4 Pharmaceutical Chemistry Department, Faculty of Pharmacy, Ahram Canadian University, 6th of October City 12566, Egypt; 5 CADD Unit, Faculty of Pharmacy, Libyan International Medical University, Benghazi 16063, Libya; radwan.alnajjar@uob.edu.ly; 6 Department of Chemistry, Faculty of Science, University of Benghazi, Benghazi 16063, Libya; 7 Department of Chemistry, University of Cape Town, Rondebosch 7700, South Africa; 8 Department of Pharmaceutical Medicinal Chemistry & Drug Design, Faculty of Pharmacy (Boys), Al-Azhar University, Cairo 11884, Egypt

**Keywords:** ureidocoumarin, DDR1/2 kinases, drug repurposing, antiproliferative activity, molecular docking, MD simulations

## Abstract

Discoidin domain receptor 1 (DDR1) kinase has emerged as a promising target for cancer therapy, and selective DDR1 inhibitors have shown promise as effective therapeutic candidates. Herein, we have identified the first coumarin-based selective DDR1 inhibitors via repurposing of a recent series of carbonic anhydrase inhibitors. Among these, ureidocoumarins **3a**, **3i**, and **3q** showed the best DDR1 inhibitory activities. The *m*-trifluoromethoxy phenyl member **3q** potently inhibited DDR1 with an IC_50_ of 191 nM, while it showed less inhibitory activity against DDR2 (IC_50_ = 5080 nM). **3q** also exhibited favorable selectivity in a screening platform with 23 common off-target kinases, including BCR-ABL. In the cellular context, **3q** showed moderate antiproliferative effects, while **3i**, with the third rank in DDR1 inhibition, exerted the best anticancer activity with sub-micromolar GI_50_ values over certain DDR1-dependent cell lines. Molecular docking and MD simulations disclosed the putative binding mode of this coumarin chemotype and provided insights for further optimization of this scaffold. The present findings collectively supported the potential improvement of ureidocoumarins **3i** and **3q** for cancer treatment.

## 1. Introduction

Among the most critical members of tyrosine kinases are the discoidin domain receptors (DDRs). So far, two types of DDRs are well-defined: DDR1 and DDR2 [1]. The two types differ in the site at which they are expressed. DDR1 is mainly present in epithelial cells, while DDR2 is predominant in connective tissue cells. Collagens activate the two DDR receptors, the feature that characterizes them over other tyrosine kinase family members. Following their activation, DDRs control a number of vital cellular behaviors, including cell differentiation and proliferation [2,3]. A growing body of literature reports proved that DDRs are key contributors among the cancer-causing factors [4,5]. Levels of either DDRs or their mutative forms were found to be overexpressed in the primary tumor tissues [6]. However, they are believed to be linked to various cancer types, such as leukemia, breast, and lung cancer [2]. Therefore, DDRs have been emerged as promising molecular targets to combat cancer and tumor progressions [7].

**Figure 1:**
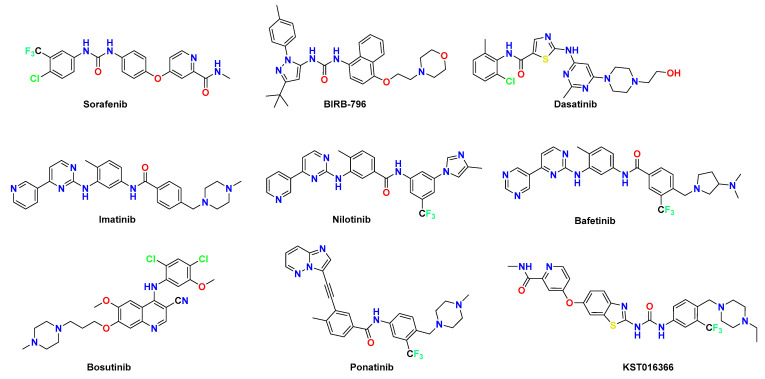
Examples of FDA-approved drugs/investigational agents as DDR1/2 kinase inhibitors.

Meanwhile, a considerable number of Food and Drug Administration (FDA)-approved drugs/investigational agents have been reported to possess DDR1/2 inhibitory effects (Figure 1). Almost all of these inhibitors exert their action upon binding to the conserved adenosine triphosphate (ATP)-binding region [8,9,10]. Therefore, these compounds lack selectivity towards their intended target due to the unplanned off-target binding to other kinases’ homologically similar ATP regions. DDR inhibitors’ non-selectivity was clear in observing the biological activity of the rapidly accelerated fibrosarcoma (RAF)/vascular endothelial growth factor receptor (VEGFR) dual inhibitor, sorafenib, as it inhibited both types of DDRs in low IC_50_ values [11]. However, the pan-p38 MAPK inhibitor, doramapimod (BIRB-796), inhibited DDR1 and DDR2 with nanomolar K*_d_* values [12]. Dasatinib, imatinib, and nilotinib were reported to act as DDR inhibitors besides their main action as breakpoint cluster region Abelson (BCR-ABL) inhibitors [13]. Moreover, bafetinib, bosutinib, and ponatinib, classified as type II BCR-ABL inhibitors, possessed remarkable DDR inhibitory effects [14,15,16,17]. In addition, KST016366, a multikinase inhibitor discovered by our group, showed potent DDR1 inhibitory activity with an IC_50_ value of 26.5 nM [18]. Nevertheless, kinase selectivity remains an issue for the aforementioned compounds. Thus, discovering DDR1 or DDR2 selective inhibitors constitutes a critical challenge facing medicinal chemists.

**Figure 2:**
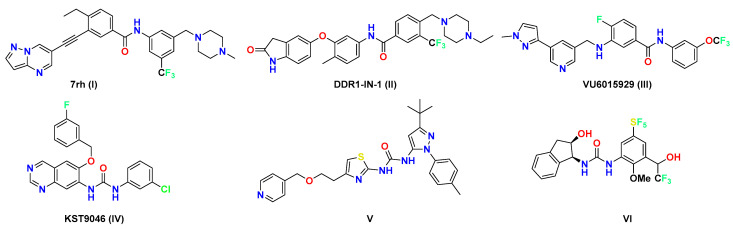
Examples of amide and urea chemotypes as selective DDR1/2 kinase inhibitors.

Over the past decade, several selective DDR1/2 inhibitors were reported with variable selectivity profiles (Figure 2). The diarylamides **7rh** (**I**) and **DDR1-IN-1** (**II**) are among the first chemotypes identified as selective DDR1 inhibitors with IC_50_ values of 6.81 and 105 nM, respectively [19,20]. **VU6015929** (**III**) is another amide DDR1/2 inhibitor featuring pyridine as a hinge binding motif with good kinome selectivity [21]. Besides amides, the quinazoline urea **KST9046** (**IV**) showed a selective DDR1 inhibitory effect with moderate potency (IC_50_ value = 4.38 μM) [22]. Later, the ureido compounds **V** and **VI** were reported as selective DDR2 and DDR1 inhibitors, respectively, with nanomolar IC_50_ values [23,24]. In addition, two patents by Nishio et al. highlighted the importance of the urea motif in DDR1 inhibition, especially those ureides with bicyclic motifs [25,26]. Similarly, certain ureido derivatives were discovered by Astex Pharmaceuticals as selective DDR1/2 inhibitors using a fragment-based drug design approach [27].

**Figure 3:**
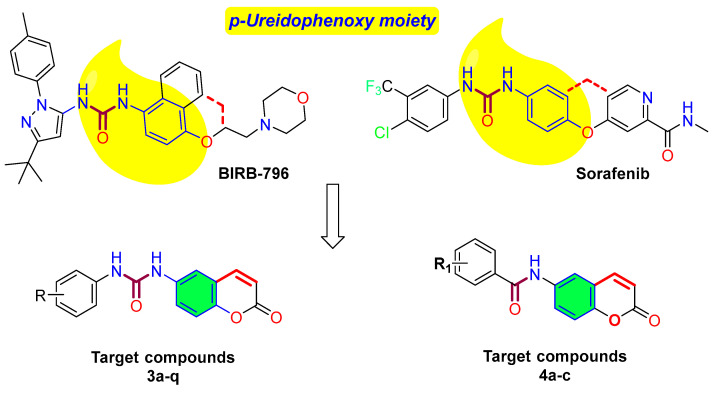
The rationale for considering **3a–q** and **4a–c** as potential DDR1/2 inhibitors.

Drug repurposing is one of the promising strategies that perhaps minimizes the cost and time consumption of constructing a new drug candidate [28]. It depends upon identifying new medicinal uses of an already approved or even an under-clinical trial drug that may ensure sufficient safety during the drug’s biological testing [29]. In the current study, we aimed to evaluate the DDR1/2 inhibitory effects of our previously reported 6-ureido/amidocoumarins carbonic anhydrase (CA) inhibitors [30]. The key reason for repurposing these ureido/amidocoumarins was their structure resemblance with the non-selective DDRs inhibitors, sorafenib and BIRB-796, particularly in the *p*-ureidophenoxy moiety (Figure 3). We hypothesized that conserving such structural features along with ring closure as αpyrone might achieve DDR1/2 inhibitory effects with a certain extent of selectivity among other kinases. Moreover, evaluating a diverse substituent on the terminal phenyl moiety was considered to outline a reliable structure–activity relationship (SAR). In particular, we added to our test library ureidocoumarin with *m*-OCF_3_ substituted phenyl group, based on the promising DDR1/2 inhibitory activity of certain inhibitors bearing such motif like VU6015929 [21] and other candidates [31,32].

## 2. Results

### 2.1. Chemistry

**Scheme 1:**
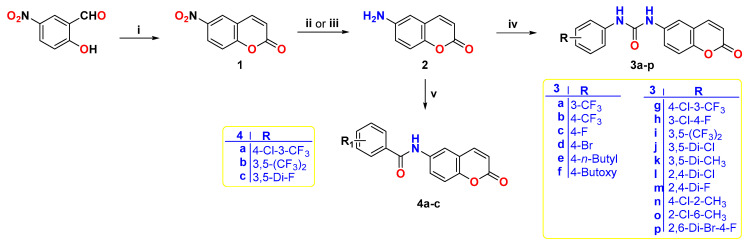
Reagents and reaction conditions: (**i**) Acetic anhydride, polyphosphoric acid, DMF, 145 °C, 6 h, 65%; (**ii**) SnCl_2_.2H_2_O, ethanol, reflux, 2 h, 71%; (**iii**) Fe powder, AcOH:EtOH:water (1:3:2 *v*/*v*), 40 °C, 1 h, 83%; (**iv**) aryl isocyanate, acetonitrile, rt, 2–18 h, DCM, rt, 18 h, 65–95%; (**v**) benzoic acid derivative, DIPEA, HATU, DMF, rt, 18 h, 25–80%.

The target ureidocoumarins **3a–p** and **4a–c** were synthesized using 6-aminocoumarin **2** as the main component, as shown in Scheme 1. First, 2-hydroxy-5-nitrobenzaldehyde was converted to 6-nitrocoumarin **1** [33] by reacting it with acetic anhydride in a mixture of polyphosphoric acid (PPA) and dimethylformamide (DMF) at 145 °C. Then, **1** was reduced to the corresponding amine **2** in good yield by either iron powder in a solution of AcOH:EtOH:water [34] or SnCl_2_ dihydrate in ethanol [35]. Next, amine **2** was treated with the suitable aryl isocyanate in acetonitrile under argon to produce the 6-ureidocoumarins **3a–p**. Coupling amine **2** with the proper benzoic acid derivative was accomplished using hexafluorophosphate azabenzotriazole tetramethyl uronium (HATU) and N,N-diisopropylethylamine (DIPEA, Hünig’s base) in anhydrous DMF to afford the desired 6-amidocoumarins **4a–c**.

**Scheme 2:**
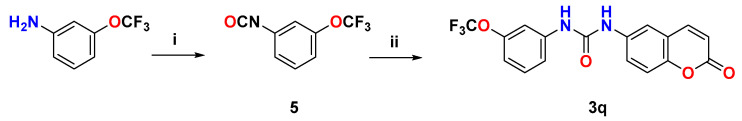
Reagents and reaction conditions: (**i**) Triphosgene, THF, 70 °C, 0.5 h; (**ii**) **2**, acetonitrile, rt, 1 h, 43%.

The ureidocoumarin **3q** was synthesized via two steps. First, the 3-(trifluoromethoxy)aniline was treated with triphosgene to produce the corresponding isocyanate, which was readily reacted with 6-aminocoumarin **2** to afford **3q** (Scheme 2).

### 2.2. In Vitro Biochemical DDR1/2 Kinase Inhibitory Activities

All compounds in this study were tested against DDR1 and DDR2 at a 10 µM dose at Reaction Biology Corporation (RBC, Malvern, PA, USA) employing the radiometric HotSpot Kinase assay protocol (Table 1). As revealed from the data, most of the compounds exhibited higher selectivity toward DDR1 than DDR2, as indicated by their percent enzymatic inhibition (%EI). The ureidocoumarins **3g** and **3i** showed better DDR1/2 inhibitory activity than the corresponding amide congeners **4a** and **4b**. The 3,5-Bis-trifluoromethylphenyl moiety in **3i** and **4b** is favorable for DDR inhibition rather m-trifluoromethyl-p-chlorophenyl in **3g** and **4a**. Among the 6-ureidocoumarins, the mono-substituted m-trifluoromethylphenyl derivative **3a** (DDR1; %EI = 92.65, DDR2; %EI = 83.63) and m-trifluoromethoxyphenyl member **3q** (DDR1; %EI = 87.37, DDR2; %EI = 70.07) showed the best DDR1/2 inhibitory activity, being superior to the 3,5-bis-trifluoromethylphenyl member **3i** (DDR1; %EI = 77.06, DDR2; %EI = 67.34). 

While comparing the positional isomers **3a** and **3b**, it was found that shifting the –CF_3_ moiety from meta to para-position of the phenyl ring abolished the DDR inhibitory potency. These biochemical outcomes indicate that either trifluoromethyl or trifluoromethoxy groups at the meta-position of phenyl are critical for achieving the DDR inhibitory effect. This may be attributed to their contribution to crucial hydrophobic interactions with certain relevant residues in the DDR1/2 kinase domain. Referring to the dichlorophenyl derivatives **3j** and **3l**, it is evident that 3,5-disubstitution with chlorine was more favorable for DDR inhibition than 2,4-disubstitution. Replacing the 3,5-dichlorophenyl (**3j**; %EI = 48.53 and 23.33 against DDR1 and DDR2, respectively) with 3,5-dimethylphenyl (**3k**; %EI = 33.76 and 17.29) resulted in reduced DDR inhibitory potency. Moreover, the 3,5-CF_3_ chemotype **3i** elicited a more remarkable DDR1/2 inhibition than its corresponding dimethyl analog **3k**, emphasizing the indispensable nature of –CF_3_ moiety in DDR1/2 inhibition.

Upon comparing the relevant pairs, the 2,4-dichlorophenyl member (**3l**; %EI = 19.04 and 17.13 against DDR1 and DDR2, respectively) displayed superior activity to its 2,4-difluorophenyl homologue (**3m**; %EI = 5.83 and 0.78). The chloro-substituted tolyl derivatives **3n** and **3o** exerted comparable activity, revealing that the insertion of chlorine at the 2- or 4-position of the tolyl ring has a similar impact on DDR1/2 inhibition. The same was applied for the p-oxybutyl and p-butyl ureidocoumarins, **3f** and **3e**, respectively.

Compounds **3a**, **3g**–**j**, and **3q**, which showed considerable activity in the single-dose assay, were advanced further to obtain their IC_50_ values against DDR1 and DDR2 kinases (Table 2). The pan-kinase inhibitor **staurosporine** along with the previously reported DDR1 inhibitors **DDR1-IN-1** and **VU6015929** were used as reference compounds. The 3-trifluoromethoxyphenyl derivative **3q** stood out as the best among the tested members in terms of both potency and DDR1 selectivity. **3q** potently inhibited DDR1 with a IC_50_ value of 0.191 ± 0.018 µM *versus* a 5.08 ± 0.181 µM value regarding DDR2, with selectivity index (SI) of 26.6. This differential inhibitory activity of **3q** toward DDR1 offers merit over the previously reported non-selective inhibitors **DDR1-IN-1** and **VU6015929**. Moreover, the 3-trifluoromethylphenyl ureide **3a** elicited selective DDR1 suppressive activity (IC_50_ = 0.321 µM) than DDR2 (IC_50_ = 2.39 µM) with SI value of 7.5 The same selectivity trend was noticed with the 3,5-bis-CF_3_ member **3i** (SI = 12.4). Besides these top three derivatives featuring –CF_3/_–OCF_3_, the other compounds manifested moderate DDR1 inhibitory activity (IC_50_ = 1.66–2.90 µM) along with weak effects toward DDR2 (IC_50_ > 10 µM).

### 2.3. Kinase Profile of Representative Compounds

#### 2.3.1. Prediction of Kinase Targets by KinScreen

**Figure 4:**
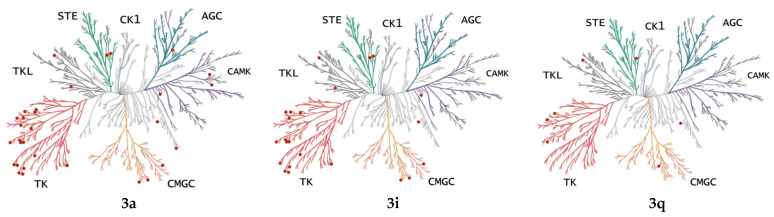
KinScreen prediction of compounds **3a**, **3i**, and **3q** with minimal confidence set as 0.2, illustration reproduced courtesy of Cell Signaling Technology, Inc. (www.cellsignal.com) (accessed on 10 February 2024).

To acquire insights into the kinase profile of this array of ureidocoumarins, the top three potent candidates, **3a**, **3i**, and **3q,** were subjected to the KinScreen library, which encompasses all different classes of kinases [36]. KinScreen allows the prediction of kinase targets with PASS software (https://www.way2drug.com/KinScreen/, accessed on 10 February 2024) by searching for analogous compounds across the ChEMBL database to unravel the molecular mechanism of action of the compound. Also, the results are visualized on the kinome tree to assess the distribution of targets across kinase families. As illustrated in Figure 4, compounds **3a, 3i**, and **3q** showed sound selectivity among various kinases, particularly the *m*–OCF_3_ member **3q**. The top five sensitive kinases for each compound were listed in Table 3, along with the prediction accuracy and confidence scores. 

Interestingly, **3a** and **3i** showed similar kinase inhibitory fashion, being expected inhibitors to MAPK kinase 7, MAP kinase p38 gamma, RAF, Ephrin type-B receptor 2, and DDR2. In contrast, the **3q** kinase tendency was mainly towards RAF and TrkA. These in silico predictions may suggest a favorable selectivity profile of these examined coumarins.

#### 2.3.2. Biochemical Kinase Profile

**Figure 5:**
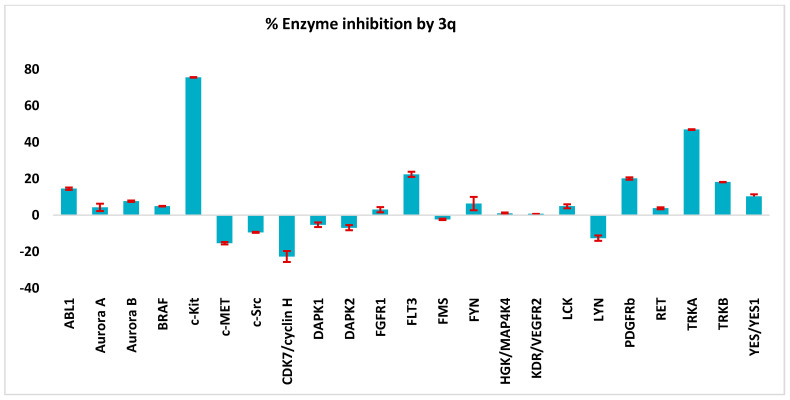
Inhibition percentages exerted by compound **3q** (10 µM) over a panel of 23 oncogenic kinases. Compound **3q** was tested in a single-dose duplicate mode, and the reactions were carried out at 10 μM ATP.

Considering the findings of preliminary KinScreen, the most potent ureidocoumarin **3q** was tested over a panel of 23 oncogenic kinases at 10 μM concentration. These tested kinases were selected based on common off targets for several DDR1/2 kinase inhibitors. As depicted in Figure 5, compound **3q** showed considerable suppressive activity against only c-Kit kinase (%EI = 75.59). Meanwhile, **3q** displayed moderate inhibitory effects (%EI = 46.95) against TrkA kinase and elicited weak activity against the rest of the tested kinases, including ABL1 kinase (the highly homologous kinase to DDR) with % inhibitions < 25.

### 2.4. In Vitro Cell-Based Antiproliferative Activities

Six members, **3a**, **3g**–**j**, and **3q**, out of the investigated compounds were further evaluated for their antiproliferative effects against human cancer cell lines, including leukemia (K562), colon cancer (HCT116), non-small cell lung (NSCL) cancer (A549, NCI-H23 and NCI-H460), and breast cancer (MCF-7 and T47D) cells. The tested cell lines’ choice depended on their elevated DDR1 expression levels. The cell lines were all treated with a 10 µM concentration of the tested compounds, and the results were expressed as the percentage of growth inhibition (%GI) (Table 4). 

Among the tested members, 3,5-disubstituted-phenylureide derivatives **3i** and **3j** exhibited the highest growth inhibitory effects. Compound **3i**, the third top member as DDR1 inhibitor, noticeably inhibited the cell growth at percentages between 84.76 and 100.22. The %GI observed by **3j** ranged from 83.76 to 106.33. Unexpectedly, the most potent DDR1 inhibitors, **3a** and **3q**, exerted moderate cytostatic activity. However, **3q** was significantly better than **3a** over all cell lines, with a %GI of 45.65–69.93. Moreover, the moderate DDR1 inhibitor **3g** showed remarkable growth inhibitory activity, particularly against NCI-H23. Similar to **3a**, compound **3h** exerted modest antiproliferative activity (% GI <35). 

This discrepancy between biochemical and cellular outcomes may stem from the variation in physicochemical properties that control the cellular potency. The physicochemical characteristics of **3a** and **3q** may be unfavorable for cell permeation and penetration, as indicated by their CLogP and molar refractivity (MR). Upon correlating the observed cellular activity of **3a**, **3g**–**j**, and **3q** with their molecular descriptors CLogP and molar MR, it was found that the anticancer activity was mainly modulated by the steric factor MR followed by the lipophilicity parameter CLogP (Table 4). Compounds **3a** and **3h** with the least MR value (84.77 and 83.27 cm^3^/mol) showed this series’ modest growth inhibitory activity. In contrast, the most cell-active ureides, **3g**, **3i**, and **3j**, possess the highest MR (87.47–91.27 cm^3^/mol) and CLogP (4.65–5.03) values. In the middle of these two extremes lies compound **3q** with moderate MR, CLogP, and anticancer activity.

In view of the promising anticancer effects of both compounds **3i** and **3j**, they were further evaluated in a five-dose testing mode to determine their GI_50_ (the molar concentration causing 50% GI). The GI_50_ values of **3i**, **3j**, and the selective DDR1 inhibitors **DDR1-IN-1** [20] and **7rh** [19] are presented in Table 5. Interestingly, **3i** elicited superior anticancer potency to **7rh** against all tested cell lines except K562. Also, **3i** showed better anticancer activity compared to DDR1-IN-1 against HCT116, A549, and T47D cell lines. Compound **3i** exerted GI_50_ values of 0.55, 0.732, 0.94, and 0.69 µM over K562, HCT116, NCI-H23, and NCI-H460 cells, respectively. The sub-micromolar potency of **3i** over these cell lines indicates the possibility of other underlying mechanisms, besides DDR1 inhibition, responsible for the antiproliferative activity of **3i** towards those cell lines. Inhibition of the tumor relevant carbonic anhydrases IX and XII may be one of these additional mechanisms [30]. Moreover, compound **3i** suppressed the growth of **DDR1-IN-1**-resistant cell lines A549 and T47D with GI_50_ values of 1.59 and 1.34 µM, respectively. The other ureidocoumarin **3j** showed reasonable potency over all tested cell lines with a GI_50_ value of 1.93–2.90 µM.

### 2.5. In Silico ADMET Prediction 

The inadequate bioavailability profile of numerous chemical compounds is a primary reason for their lack of success in clinical trials. Consequently, in silico ADMET (absorption, distribution, metabolism, excretion, and toxicity) prediction assessment has emerged as a crucial component in the drug discovery process. This evaluation serves to mitigate the probability of drug failure during clinical trials. This study predicted the drug-likeness characteristics of compounds **3i** and **3q** by employing the pkCSM server (https://biosig.lab.uq.edu.au/pkcsm/ accessed on 10 February 2024) (Table 6) [37]. 

Both **3i** and **3q** showed moderate water solubility and predicted favorable gastrointestinal absorption (>87%) with the possibility of being substrates for P-gp (P-glycoprotein). In contrast, **3i** and **3q** displayed poor Blood-Brain Barrier (BBB) permeability. In terms of metabolism, ureidocoumarins **3i** and **3q** displayed CYP1A2, CYP2C9, and CYP2C19 inhibitory potential, whereas **3i** was a non-inhibitor of CYP2D6 and CYP3A4. Regarding the excretion parameter of renal OCT2, both **3i** and **3q** were non-substrate to renal OCT2. 

The issue of toxicity is an essential factor within the field of drug research. Although a chemical may demonstrate effectiveness and be considered as having therapeutic benefits, its potential toxicity may impede its practical application. The bacterial reverse mutation test (AMES) for toxicity profiles molecules with potential mutagenic tendencies. Both compounds **3i** and **3q** are negative, indicating that they are not predicted to act as mutagens or carcinogens. The maximum recommended tolerated dose (MRTD) provides an estimate of the toxic dose threshold of chemicals in humans. Dose-related toxicity is associated with MRTD > 0.477 log(mg/kg/day). There may be no possible dose-related toxicity as the MRTD of the compounds is ≤0.477 log(mg/kg/day). The main cause contributing to the emergence of acquired long QT syndrome, which can result in deadly ventricular arrhythmia, is the suppression of the potassium channels encoded by an ether-a-go-go-related gene (hERG). Compounds **3i** and **3q** demonstrated no inhibitory effects on the human hERG I. However, **3i** and **3q** may be triggering hepatotoxicity, and should be avoided for patients with hepatic impairment. Based on the findings of this in silico study, **3i** and **3q** showed acceptable pharmacokinetic properties along with a relatively safe toxicity profiles.

### 2.6. Molecular Docking Studies

**Figure 6:**
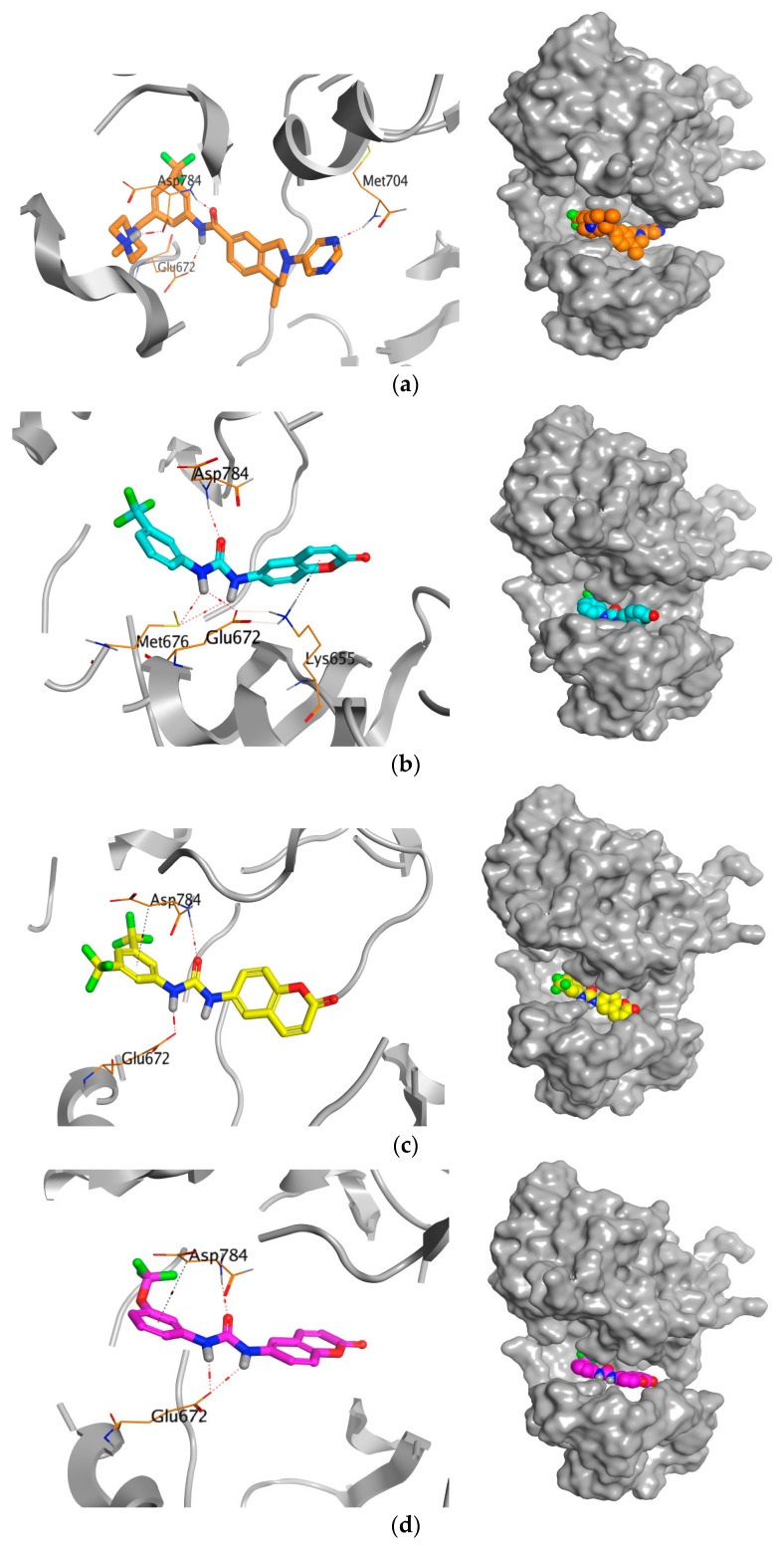
3D interactions and positioning of co-crystallized inhibitor (**Co**) (**a**), **3a** (**b**), **3i** (**c**), and **3q** (**d**) within the binding pocket of the DDR1 (PDB ID: 5FDP) target receptor.

Observing the binding pocket of the DDR1, it has appeared as an elongated cleft within the monomeric protein. The co-crystallized inhibitor (**Co**) is stabilized within its binding pocket by forming two hydrogen bonds (HBs) with Glu672 and Asp784, using its amidic linkage. Also, it formed one HB with Asp784 through its protonated piperidine moiety and one HB with Met704 in the hinge region through its pyrimidine nucleus (Figure 6a). Therefore, it was obvious that Glu672, Asp784, and Met704 amino acids’ binding is very crucial for producing the inhibitory activity towards the DDR1 receptor. It is worth mentioning that the docked **Co** achieved a binding score of −10.52 kcal/mol (RMSD = 1.66 Å). 

In addition, all the examined candidates showed promising binding scores and modes, especially the urea featuring derivatives, which agrees with the biological findings of DDR1 inhibitory activities. The most active compounds (**3a**, **3i**, and **3q**) were selected for further investigation compared to the **Co**. Compound **3a** (Score = −7.08 kcal/mol, RMSD = 1.16 Å) was engaged with Glu672 and Asp784 by two HBs via its urea spacer. In addition, it also bound Met676 with two HBs using its urea moiety. Further, it bound Lys655 with a pi-cation interaction (Figure 6b). However, compound **3i** (Score = −7.62 kcal/mol, RMSD = 1.48 Å) formed two HBs with Glu672 and Asp784 through its urea linkage. Also, it bound Asp784 with an extra pi-HB using its terminal substituted phenyl ring (Figure 6c). On the other hand, the **3q** candidate (Score = −7.40 kcal/mol, RMSD = 1.67 Å) showed three HBs with Glu672 (2) and Asp784 (1) through its urea linkage, and it bound Asp784 with an additional pi-HB using its terminal substituted phenyl ring (Figure 6d). 

Accordingly, based on the similar binding modes of **3a**, **3i**, and **3q** to the crucial amino acids of the DDR1 receptor pocket compared to the **Co**, they are highly recommended to act as promising DDR1 inhibitors. Also, their binding modes clarified the importance of their urea moiety to interact with the target receptor’s two important amino acids (Glu672 and Asp784). 

Notably, it is worth mentioning that one of the 3,5-disubstituted CF_3_ groups at the phenyl ring of compound **3i** formed a steric clash at the binding pocket of the DDR1 receptor. This may explain the lower binding interactions of **3i** within the DDR1 binding pocket compared to both **3a** and **3q** members, as represented above. On the other side, the 3-OCF_3_ substituent of the phenyl ring of **3q** achieved superior fitting compared to the 3-CF_3,_ one of the phenyl rings of **3a** within the terminal pocket of the DDR1 receptor. This was confirmed by the frontier binding mode of the **3q** candidate, which formed three HBs and one pi-H interaction with the two essential amino acids of the DDR1 receptor (Glu672 and Asp784) as indicated by the binding mode of **Co**. Accordingly, we could investigate the great importance of the additional oxygen atom in the 3-OCF_3_-substituted phenyl ring of compound **3q** in producing the perfect fit to the DDR1 binding site compared to the corresponding 3-CF_3_-substituted phenyl of **3a**. This explains greatly the superior DDR1 inhibitory potential of compound **3q** compared to both **3a** and **3i** members, as discussed in the biological data section. 

### 2.7. Molecular Dynamics (MD) Simulations 

This was performed to study the exact behavior of the studied candidates within the target DDR1 receptor (PDB ID: 5FDP) throughout the simulation time (200 ns) in comparison to the co-crystallized antagonist (**Co**).

#### 2.7.1. RMSD Analysis

**Figure 7:**
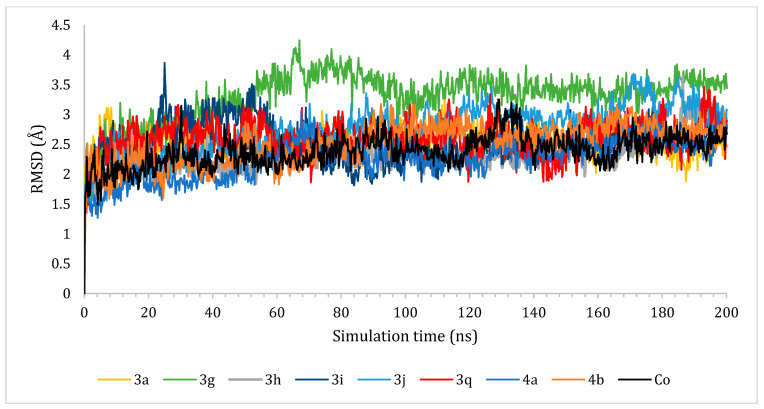
The RMSD of complexes (**3a**, **3g–j, 3q**, **4a**, **4b**, and **Co**) for the DDR1 binding site (PDB ID: 5FDP) as a function of simulation time (200 ns).

The Root-Mean-Square Deviation (RMSD) is essential to quantitatively study the degree of each complex deviation compared to its initial position. This could evaluate the overall stability of the system throughout the simulation time. The obtained RMSD of all complexes are plotted in Figure 7. As it can seen in Figure 7, all complexes showed a stable RMSD of around 3.00 Å, which indicates that the protein did not undergo any conformational change within its structure. 

**Figure 8:**
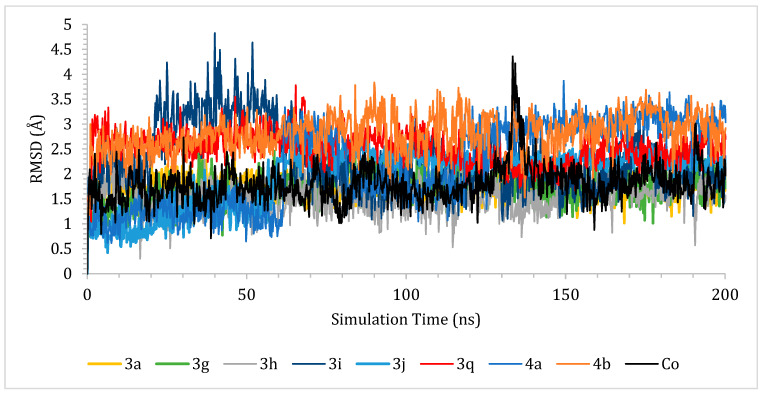
The RMSD of ligands (**3a**, **3g**–**q**, **4a**, **4b**, and **Co**) for the DDR1 binding site (PDB ID: 5FDP), respectively, as a function of simulation time (200 ns).

Observing the primary position of each examined ligand within the binding pocket of DDR1, the ligand RMSD was studied as a function of the simulation time (200 ns), Figure 8. All ligands showed moderate fluctuations below 4 Å except for **3i**, which approached 4.5 Å due to higher fluctuations within, at around 25–55 ns. Notably, the superior DDR1 inhibitors from the biological studies (**3a**, **3i**, and **3q**) showed greatly similar or better behaviors within the target receptor compared to that of the reference **Co**. Compound **3i** did not leave the receptor pocket throughout the 200 ns of the simulation time and showed moderate fluctuations from 1.5 to 3.7 Å. In addition, **3a** and **3q** represented lower fluctuations within (1–2.4) and (1.8–3.6) Å. Also, Compound **3q** did not get outside the receptor pocket throughout the 200 ns of the simulation time, indicating a very promising affinity. Again, this confirms the importance of the 3-OCF3 substituent of the phenyl ring **3q** in achieving superior fitting within the target DDR1 receptor pocket.

#### 2.7.2. Histogram and Heat Map Analysis

**Figure 9:**
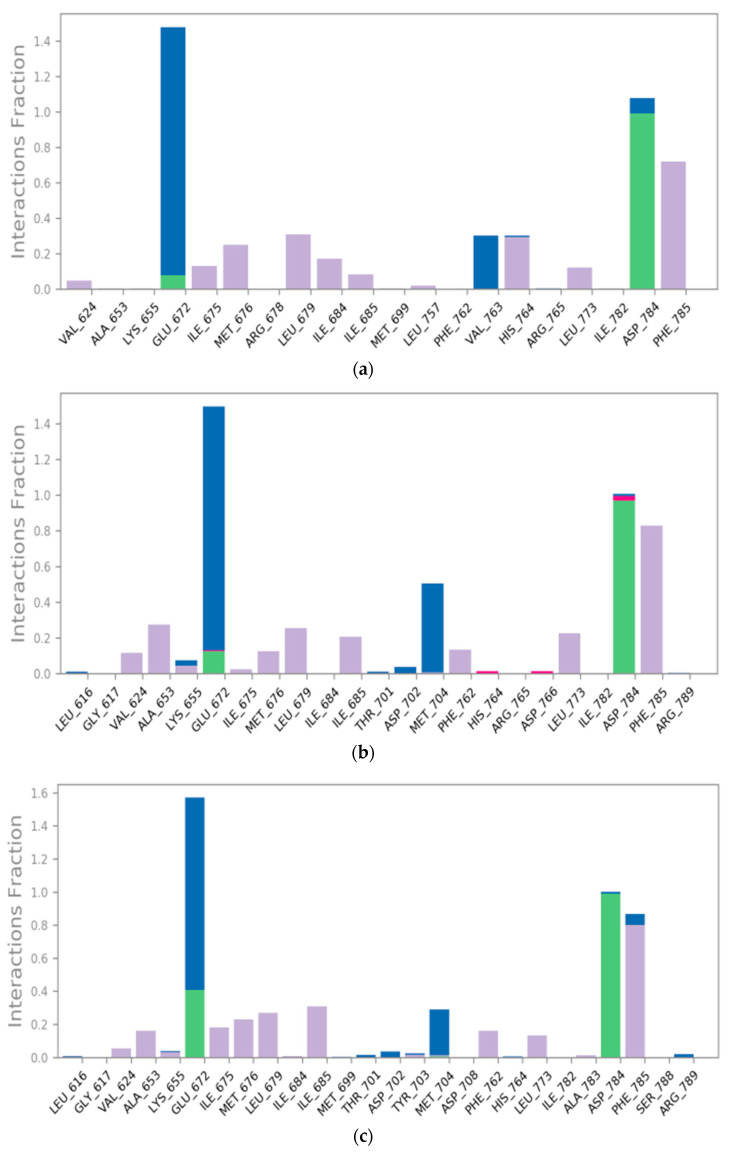

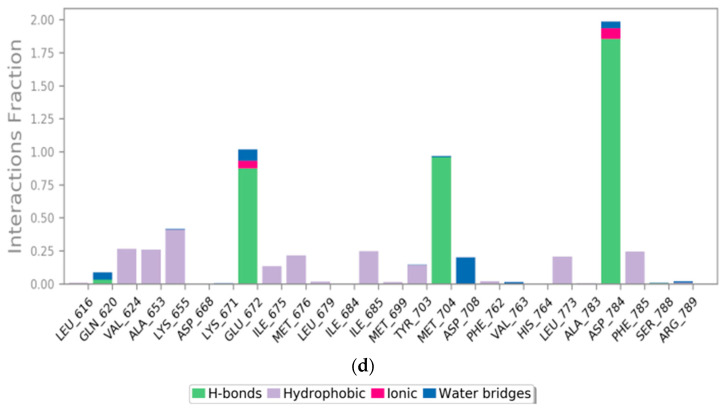
Histogram describing the binding interactions between the DDR1 protein (PDB ID: 5FDP) and its ligand during the simulation time of 200 ns for (**a**) **3a**, (**b**) **3i**, (**c**) **3q**, and (**d**) **Co**.

The histogram describes the types and percentages of the target receptor amino acids’ interactions with the binding ligands. The previously mentioned superior complexes (**3a**-5FDP, **3i**-5FDP, and **3q**-5FDP) are described in Figure 9 and compared to the reference (**Co**-5FDP). The histograms for all the examined complexes (**3i**-5FDP, **3a**-5FDP, **3q**-5FDP, and **Co**-5FDP) showed that Glu672, Asp784, Phe785, and Met704 were the most crucial amino acids contributing to the interactions. Their contribution percentages were found to be (150, 100, 80, and 50%) for **3a**-5FDP (Figure 9b), (150, 110, 80, and 0%) for **3i**-5FDP (Figure 9a), (160, 100, 90, and 30%) for **3q**-5FDP (Figure 9c), and (110, 200, 25, and 100%) for **Co**-5FDP (Figure 9d), respectively. 

The types of interactions for **3a**-5FDP (Figure 9b) were found to be H-bonds, ionic bonds, and water bridges for Glu672 and Asp784, hydrophobic interactions for Phe785, and water bridges for Met704. However, the types of interactions for **3i**-5FDP (Figure 9a) were represented as H-bonds, water bridges for Glu672 and Asp784, and hydrophobic interactions for Phe785. Moreover, those of **3q**-5FDP (Figure 9c) were shown as H-bonds and water bridges for Glu672 and Asp784, hydrophobic interactions and water bridges for Phe785, and water bridges for Met704. Furthermore, the described types of interactions for **Co**-5FDP (Figure 9d) were H-bonds, ionic bonds, and water bridges for Glu672 and Asp784, hydrophobic interactions for Phe785, and H-bonds and water bridges for Met704. 

Collectively, the histograms of **3a**-5FDP, **3i**-5FDP, **3q**-5FDP, and **Co**-5FDP confirmed that Glu672, Asp784, Phe785, and Met704 are the most critical amino acids contributing to the interactions and consequentially are responsible for the produced antagonistic activity as well.

Figure S2 (SI) shows the heat maps for the studied complexes (**3a**-5FDP, **3i**-5FDP, **3q**-5FDP, and **Co**-5FDP), which describe the most important amino acids of the DDR1 receptor contributing to the interactions throughout the simulation time (200 ns). 

The heat map of **3a**-5FDP clarified that Glu672, Asp784, and Phe785 interactions were distributed equally over 200 ns of the simulation time (Figure S2a). For **3i**-5FDP, Glu672 contributions were all over the simulation time except from (170 to 180) ns, where the interactions were significantly decreased. In addition, the contributions of both Asp784 and Phe785 were distributed equally throughout the 200 ns of the simulation time. However, the Met704’s contributions were weak and started after 25 ns to the end of the simulation time (Figure S2b). However, the heat map of **3q**-5FDP represented that the contributions of Glu672, Asp784, and Phe785 were all over the simulation time and increased gradually after 10 ns to the end of the simulation. Finally, **Co**-5FDP’s heat map showed that Glu672 and Asp784 contributed to the interactions all over the 200 ns of the simulation. Also, Met704 showed the same behavior except from 130 to 135 ns, where its contributions nearly disappeared. Again, the heat maps of **3a**-5FDP, **3i**-5FDP, **3q**-5FDP, and **Co**-5FDP confirmed that Glu672, Asp784, Phe785, and Met704 are the ones responsible for the interactions throughout the 200 ns of the simulation and the produced DDR1 inhibition is mainly attributed for their presence.

Moreover, the covalent, Coulomb, lipophilic, hydrogen-bonding, generalized Born electrostatic solvation, and Van der Waals energies were calculated (Table 7) using the thermal_mmgbsa.py python script of Schrodinger.

According to Table 7, compound **3i** was the most promising, with a ΔG binding energy of −67.76 Kcal/mol, followed by **3q** and **3a** (−63.68 and −59.46) Kcal/mol, respectively. These values were promising compared to that of the **Co** (−79.82 Kcal/mol) as a reference. Moreover, the bind packing energies for **3i**, **3a**, and **3q** (−1.84, −1.94, and −1.59) Kcal/mol, respectively, were superior to that of the **Co** (−0.56 Kcal/mol), indicating promising fitting for the examined candidates within the DDR1 receptor pocket. This outcome confirms the recommended potential inhibitory activities of **3i**, **3a**, and **3q** compounds towards the DDR1 receptor.

## 3. Materials and Methods

### 3.1. Chemistry

General: The solvents and reagents utilized in this study were purchased from commercial suppliers and employed without additional purification. The reaction progress was observed by thin-layer chromatography (TLC) using a silica gel 60 F254 TLC plate manufactured by Merck. ^1^H and ^13^C NMR spectra were obtained using a Bruker Advance 400 MHz spectrometer, with deuterated dimethylsulfoxide (DMSO) as the solvent. Chemical shifts (δ) are expressed in parts per million (ppm) relative to the internal standard tetramethylsilane (TMS). The abbreviations s, d, t, and m are commonly used to denote singlet, doublet, triplet, and multiplet, respectively. The reported units for coupling constants (*J*) are hertz (Hz). The acquisition of high-resolution mass spectra (HRMS) was performed using either the Waters Acquity UPLC/Synapt G2 QTOF MS or the JMS 700 mass spectrometer manufactured by Jeol, Japan. The starting material **1** [33] and key intermediate **2** [34,35] were prepared adopting the reported procedure.

#### 3.1.1. Synthesis of 6-Ureidocoumarins **3a**–**p** and 6-Amidocoumarins **4a**–**c**

These titled compounds have been synthesized from compound **2** and fully characterized as previously reported [30].

#### 3.1.2. Synthesis of 1-(2-Oxo-2*H*-chromen-6-yl)-3-(3-(trifluoromethoxy)phenyl)urea (**3q**)

Triphosgen (40 mg, 0.135 mmol) was added to a solution of 3-(trifluoromethoxy)aniline (72 mg, 0.407 mmol) in tetrahydrofuran (10 mL). The reaction mixture was stirred at 70 °C for 30 min under an argon atmosphere. The solvent was distilled off under reduced pressure, and the obtained residue was treated with a solution of 6-aminocoumarin (65.5 mg, 0.407 mmol) in acetonitrile (3 mL). The reaction mixture was stirred at room temperature for 1 h. The solvent was removed under vacuum, and the residue was purified by column chromatography using a mixture of hexane and ethyl acetate (3:1, then switching to 2:1 and finally 3:1 *v*/*v*) to afford the titled compound in pure form: a white solid with a yield of 43%; ^1^H NMR (400 MHz, DMSO-*d*_6_) δ 9.07 (s, 1H), 8.97 (s, 1H), 8.09 (d, *J* = 9.6 Hz, 1H), 7.92 (d, *J* = 2.5 Hz, 1H), 7.73 (s, 1H), 7.57 (dd, *J* = 8.9, 2.6 Hz, 1H), 7.43–7.35 (m, 2H), 7.31 (d, *J* = 8.8 Hz, 1H), 6.95 (d, *J* = 8.2 Hz, 1H), 6.49 (d, *J* = 9.5 Hz, 1H); ^13^C NMR (100 MHz, DMSO-*d*_6_) δ 160.5, 153.0, 149.3, 149.2, 144.8, 141.9, 136.3, 130.8, 123.3, 120.6 (q, *J* = 254 Hz), 119.3, 117.4, 117.3, 117.1, 117.0, 114.3, 110.7; HRMS (EI) *m*/*z* calculated for C_17_H_12_F_3_N_2_O_4_ [M+H]^+^ was 365.0749, found 365.0742.

### 3.2. Biological Evaluation

#### 3.2.1. In Vitro Kinase Assay

Reaction Biology Corporation (RBC) Kinase HotSpot^SM^ service was adopted for biochemical evaluation of the target compounds against DDR1 and DDR2 kinases along with the kinase profile of compound **3q** according to the reported assay protocol [38,39].

#### 3.2.2. Cell-Based Anticancer Evaluation

The evaluation of the antiproliferative activities of compounds **3a**, **3g**–**j**, and **3q** over a panel of DDR1-dependent cell lines was conducted employing the sensitive Sulforhodamine B (SRB) assay at the National Cancer Institute (NCI), Bethesda, Maryland, USA, following the standard protocol [40].

### 3.3. In Silico Studies

#### 3.3.1. KinScreen Prediction

The prediction of potential kinase targets of compounds **3a**, **3i**, and **3q** was carried out utilizing the KinScreen online server, setting the minimum confidence as 0.2 [36].

#### 3.3.2. ADMET Prediction

The pharmacokinetic ADME properties and toxicity parameters of compounds **3i** and **3q** were studied by using the pkCSM descriptors algorithm protocol [37].

#### 3.3.3. Molecular Docking Study

The target DDR1 receptor was obtained from the Protein Data Bank (PDB ID: 5FDP) and prepared using the Protein Preparation Wizard tool of the Schrodinger suite [41]. Where water molecules were removed, hydrogen atoms were added in 3D dimensions, bond orders were assigned, and hydrogen bonding networks were optimized. Then, it was energy minimized using the Optimized Potentials for Liquid Simulation (OPLS4) force field [42]. The chemical structures of the examined ligands were sketched in ChemDraw, and their 3D structures were prepared with energy minimized by Maestro (v.12.9). For the molecular docking study, the glid software (v.12.9) was used with default settings. Figures were generated using Maestro and Chimera [43].

#### 3.3.4. Molecular Dynamics (MD) Simulations

The desmond package of Schrödinger LLC and its thermal_mmgbsa.py python script [44] were used to apply the MD simulations and the Molecular Mechanics Generalized Born Surface Area (MM-GBSA) energies [45] for the examined complexes, respectively. The detailed methods were described in the Supplementary Data (SI 1 and 2).

## 4. Conclusions

In the present study, we have applied a drug repurposing approach for a recent array of carbonic anhydrase inhibitors and successfully identified new coumarin-based selective DDR1 inhibitors. The *m*-CF_3_/OCF_3_ featuring ureidocoumarins **3a**, **3i,** and **3q** elicited the best DDR1 inhibitory effects. The *m*-trifluoromethoxy phenyl derivative **3q** was the most potent DDR1 inhibitor with an IC_50_ of 191 nM and a SI of 26.6. The kinase profile of **3q** revealed its good kinome selectivity. Despite the promising biochemical outcomes of **3a** and **3q**, they exerted moderate anticancer potency against a set of DDR1-overexpressing cell lines, probably due to their physicochemical properties. The 3,5-bis-trifluoromethyl phenyl member **3i** elicited distinct anticancer activity, outperforming the selective DDR inhibitors **7rh (I)** and **DDR1-IN-1 (II)**. Considering the results of both cell-free and cell-based assays, the ureidocoumarins reported herein may possess additional mechanisms of action besides their DDR1 inhibition that contribute to their anticancer activity. Molecular docking and MD simulations provided insights into the putative binding mode of such coumarin chemotype. Both compounds **3i** and **3q** may serve as promising starting points for the further optimization of potent DDR1 inhibitors as anticancer agents.

## Figures and Tables

**Table 1:** Inhibitory effects of compounds **3a–q** and **4a–c** against DDR1 and DDR2 kinases at 10 µM ^a^.

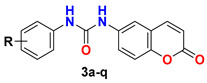		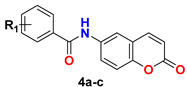	
Compound	R	% Inhibition at 10 μM (SEM)	
		DDR1	DDR2
**3a**	3-CF_3_	92.65 (0.50)	83.63 (0.34)
**3b**	4-CF_3_	24.49 (1.29)	11.67 (4.48)
**3c**	4-F	25.60 (0.68)	15.95 (6.69)
**3d**	4-Br	23.05 (2.12)	7.53 (1.95)
**3e**	4-n-Butyl	15.36 (0.34)	3.07 (1.41)
**3f**	4-Butoxy	10.55 (0.77)	5.52 (2.69)
**3g**	4-Cl-3-CF_3_	48.96 (0.82)	39.26 (9.89)
**3h**	3-Cl-4-F	57.20 (2.30)	27.47 (0.23)
**3i**	3,5-CF_3_	77.06 (0.55)	67.34 ± (1.41)
**3j**	3,5-Cl	48.53 (1.17)	23.33 (1.49)
**3k**	3,5-CH_3_	33.76 (0.59)	17.29 (1.97)
**3l**	2,4-Cl	19.04 (1.39)	17.13 (1.07)
**3m**	2,4-F	5.83 (3.72)	0.78 (5.17)
**3n**	4-Cl-2-CH_3_	10.70 (1.13)	8.02 (3.85)
**3o**	2-Cl-6-CH_3_	15.69 (5.59)	−1.40 (5.19)
**3p**	2,6-Br-4-F	15.02 (0.38)	17.06 (0.40)
**3q**	3-OCF_3_	87.37 (1.05)	70.07 (0.32)
**4a**	4-Cl-3-CF_3_	5.82 (2.16)	3.77 (3.71)
**4b**	3,5-CF_3_	12.64 (0.26)	6.06 (7.72)
**4c**	3,5-F	22.37 (0.75)	17.01 (1.89)

^a^ Compounds were tested in a single-dose duplicate mode at 10 μM, and the reactions were carried out at 10 μM ATP. The presented data are the average of a duplicate assay with standard error of the mean (SEM) in parenthesis.

**Table 2:** IC_50_ values of compounds **3a**, **3g**–**j**, and **3q** against DDR1 and DDR2 kinases ^a^.

Compound No.	(IC_50_, μM) (SEM)		
	DDR1	DDR2	SI ^b^
**3a**	0.321 (0.174)	2.39 (0.344)	7.5
**3g**	2.90 (0.077)	>10	>3.4
**3h**	1.66 (0.082)	>10	>6.0
**3i**	1.53 (0.450)	19.0 (10.9)	12.4
**3j**	2.52 (0.454)	>10	>4.0
**3q**	0.191 (0.018)	5.08 (0.181)	26.6
**Staurosporine**	0.005 (0.000)	0.0009 (0.000)	0.18
**DDR1-IN-1**	0.0747 (0.005)	0.0597 (0.004)	0.8
**VU6015929**	0.0684 (0.002)	0.00155 (0.000)	0.02

^a^ Compounds were tested in a 10-dose duplicate IC_50_ mode with 3-fold serial dilution starting at 20 μM, and the reactions were carried out at 10 μM ATP. The presented data are the average of the duplicate assay with standard error of the mean (SEM) in parenthesis ^b^ SI: Selectivity index (IC_50_ DDR2/IC_50_ DDR1).

**Table 3:** KinScreen results of compounds **3a**, **3i**, and **3q**.

Compound No.	Confidence	Name	UniProt ID	ChEMBL ID	Prediction Accuracy
**3a**	0.66	Dual specificity MAPK kinase 7	O14733	CHEMBL3530	0.69
	0.56	MAP kinase p38 gamma	P53778	CHEMBL4674	0.71
	0.51	Serine/threonine protein kinase RAF	P04049	CHEMBL1906	0.88
	0.46	Ephrin type-B receptor 2	P29323	CHEMBL3290	0.77
	0.42	Discoidin domain-containing receptor 2	Q16832	CHEMBL5122	0.84
**3i**	0.59	Dual specificity MAPK kinase 7	O14733	CHEMBL3530	0.69
	0.53	MAP kinase p38 gamma	P53778	CHEMBL4674	0.71
	0.51	Serine/threonine protein kinase RAF	P04049	CHEMBL1906	0.88
	0.47	Ephrin type-B receptor 2	P29323	CHEMBL3290	0.77
	0.45	Discoidin domain-containing receptor 2	Q16832	CHEMBL5122	0.84
**3q**	0.48	Serine/threonine protein kinase RAF	P04049	CHEMBL1906	0.88
	0.37	Nerve growth factor receptor TrkA	P04629	CHEMBL2815	0.78
	0.34	Serine/threonine protein kinase NLK	Q9UBE8	CHEMBL5364	0.78
	0.33	PDGFR receptor alpha	P16234	CHEMBL2007	0.84
	0.29	Dual specificity MAPK kinase 7	O14733	CHEMBL3530	0.69

**Table 4:** The CLogP, MR, and antiproliferative activities of compounds **3a**, **3g–j**, and **3q** against DDR1-dependent cancer cell lines at 10 µM.

Compound	CLoP ^a^	MR ^a^	% Growth inhibition at 10 µM						
			K562 ^b^	HCT116 ^c^	A549 ^d^	NCI-H23 ^d^	NCI-H460 ^d^	MCF-7 ^e^	T47D ^e^
**3a**	4.12	84.77	33.24	20.87	10.39	13.21	15.85	29.24	24.61
**3g**	4.65	89.37	80.85	84.3	60.15	95.15	81.9	88.07	91.27
**3h**	4.08	83.27	32.24	14.12	15.14	14.34	20.64	25.37	24.63
**3i**	5.03	91.27	86.27	97.91	84.76	100.22	93.64	96.19	95.2
**3j**	4.65	87.47	84.58	98.64	83.76	106.33	98.83	94.52	96.46
**3q**	4.23	87.11	67.08	66.33	45.65	56.64	69.93	57.03	69.44

^a^ CLogP and MR (molar refractivity, cm^3^/mol) values were calculated by ChemDraw Professional 16.0.1 software. ^b^ Leukemia cells. ^c^ Colon cancer cells. ^d^ Non-small cell lung (NSCL) cancer cells. ^e^ Breast cancer cells.

**Table 5:** The GI_50_ values of compounds **3i** and **3j** over DDR1-dependent cancer cells.

Compound	GI_50_, µM						
	K562 ^a^	HCT116 ^b^	A549 ^c^	NCI-H23 ^c^	NCI-H460 ^c^	MCF-7 ^d^	T47D ^d^
**3i ^e^**	0.55	0.732	1.59	0.94	0.69	1.94	1.34
**3j**	2.34	2.31	2.90	2.67	2.27	2.28	1.93
**DDR1-IN-1 ^f^**	NT ^h^	8.7	>10	NT ^h^	NT ^h^	NT ^h^	>10
**7rh ^g^**	0.038	1.13	2.74	2.08	2.98	2.15	1.88

^a^ Leukemia cells. ^b^ Colon cancer cells. ^c^ Non-small cell lung (NSCL) cancer cells. ^d^ Breast cancer cells. ^e^ The compound was tested twice by the Developmental Therapeutics Program (DTP), and the presented values are the average. ^f^ Reported data [20]. ^g^ Reported data [19]. ^h^ NT: not tested.

**Table 6:** Predicted ADMET properties of compounds **3i** and **3q**.

Properties	ADMET Properties and Values		
		3i	3q
Absorption	Water Solubility	−5.095	−4.457
	CaCO_2_ permeability	0.715	0.721
	Intestinal absorption (human)	87.169	88.852
	Skin permeability	−2.786	−2.812
	P-glycoprotein substrate	Yes	Yes
	P-glycoprotein I inhibitor	No	No
	P-glycoprotein II inhibitor	Yes	Yes
Distribution	VDss (human)	−0.471	−0.640
	Fraction unbound (human)	0.115	0.151
	BBB permeability	−0.833	−0.643
	CNS permeability	−1.561	−1.933
Metabolism	CYP2D6 substrate	No	No
	CYP3A4 substrate	Yes	Yes
	CYP1A2 inhibitor	Yes	Yes
	CYP2C19 inhibitor	Yes	Yes
	CYP2C9 inhibitor	Yes	Yes
	CYP2D6 inhibitor	No	No
	CYP3A4 inhibitor	No	Yes
Excretion	Total clearance	0.083	0.106
	Renal OCT2 substrate	No	No
Toxicity	AMES toxicity	No	No
	Max. tolerated dose (human)	0.346	0.464
	hERG I inhibitor	No	No
	hERG II inhibitor	Yes	Yes
	Oral rat acute toxicity (LD50)	2.480	2.539
	Oral rat chronic toxicity (LOAEL)	1.073	1.167
	Hepatotoxicity	Yes	Yes
	Skin sensitization	No	No
	T. Pyriformis toxicity	0.975	0.901
	Minnow toxicity	0.445	0.553

**Table 7:** Prime MM-GBSA energies for complexes (**3a**-5FDP, **3i**-5FDP, **3q**-5FDP, and **Co**-5FDP) of the DDR1 receptor.

Complex	ΔG Binding	Coulomb	Covalent	H-bond	Lipo	Bind Packing	Solv_GB	VdW	St. Dev.
**3a**	−59.46	−12.82	0.31	−0.55	−19.61	−1.94	22.80	−47.65	3.79
**3i**	−67.76	−14.25	1.08	−0.54	−20.35	−1.84	21.35	−53.20	4.44
**3q**	−63.68	−12.63	1.14	−0.87	−19.66	−1.59	20.39	−50.45	4.68
**Co**	−79.82	−27.70	2.61	−2.22	−27.51	−0.56	40.97	−65.40	5.23

Covalent: covalent binding energy; Coulomb: Coulomb energy; Lipo: lipophilic energy; H-bond: hydrogen-bonding energy; Solv_GB: generalized Born electrostatic solvation energy; VdW: Van der Waals energy; and St. Dev.: Standard deviation.

## Data Availability

Data is contained within the article.

## Supplementary Materials

The following supporting information can be downloaded at: https://www.mdpi.com/article/10.3390/ph17040427/s1. It includes ^1^H and ^13^C-NMR spectra of **3q**, HRMS chart of **3q**, Molecular dynamics simulations, DDR1/2 inhibition curves (Figure S1), and Heat maps for **3a**-5FDP, **3i**-5FDP, **3q**-5FDP, and **Co**-5FDP (Figure S2). References [46,47,48,49,50,51] are cited in the supplementary materials.
