# Supplementary material for: Identification of Ureidocoumarin-Based Selective Discoidin Domain Receptor 1 (DDR1) Inhibitors via Drug Repurposing Approach, Biological Evaluation, and In Silico Studies
Source: Pharmaceuticals (Basel). 2024 Mar 27;17(4):427. doi: 10.3390/ph17040427 (PMC11054573; doi:10.3390/ph17040427)
Supplement: Supplementary file 1 [file pharmaceuticals-17-00427-s001.zip › pharmaceuticals-2895495-supplementary.pdf]

Identification of Ureidocoumarin-Based Selective Discoidin Domain Receptor 1 (DDR1) Inhibitors via Drug Repurposing Approach, Biological Evaluation, and In Silico Studies

Ashraf K. El-Damasy [1,2,*], Hyun Ji Kim [1], Ahmed A. Al-Karmalawy [3,4], Radwan Alnajjar [5,6,7], Mohamed M. Khalifa [8], Eun-Kyoung Bang [1], Gyochang Keum [1*]

[1] *Brain Science Institute, Korea Institute of Science and Technology (KIST), Seoul 02792, Republic of Korea*

[2] *Department of Medicinal Chemistry, Faculty of Pharmacy, Mansoura University, Mansoura 35516, Egypt*

[3] *Department of Pharmaceutical Chemistry, Faculty of Pharmacy, Horus University-Egypt, New Damietta 34518, Egypt*

[4] *Pharmaceutical Chemistry Department, Faculty of Pharmacy, Ahram Canadian University, 6th of October City 12566, Egypt*

[5] *CADD Unit, Faculty of Pharmacy, Libyan International Medical University, Benghazi, 16063, Libya*

[6] *Department of Chemistry, Faculty of Science, University of Benghazi, Benghazi, 16063, Libya*

[7] *Department of Chemistry, University of Cape Town, Rondebosch 7700, South Africa*

[8] *Department of Pharmaceutical Medicinal Chemistry & Drug Design, Faculty of Pharmacy (Boys), Al-Azhar University, Cairo 11884, Egypt*

*Corresponding Authors:
ph_karem2000@mans.edu.eg , phkarem2006@gmail.com (A. K. El-Damasy), gkeum@kist.re.kr (G. Keum)

Contents **Pages**

1) ^{1}H and ^{13}C-NMR spectra of **3q** 2

2) HRMS chart of **3q** 3

3) Molecular dynamics simulations 4–5

4) DDR1/2 inhibition curves 6–9

5) Heat maps for **3a**-5FDP, **3i**-5FDP, **3q**-5FDP, and **Co**-5FDP 9–11

1) ^{1}H and ^{13}C-NMR spectra of 3q

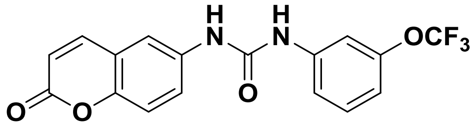

3q

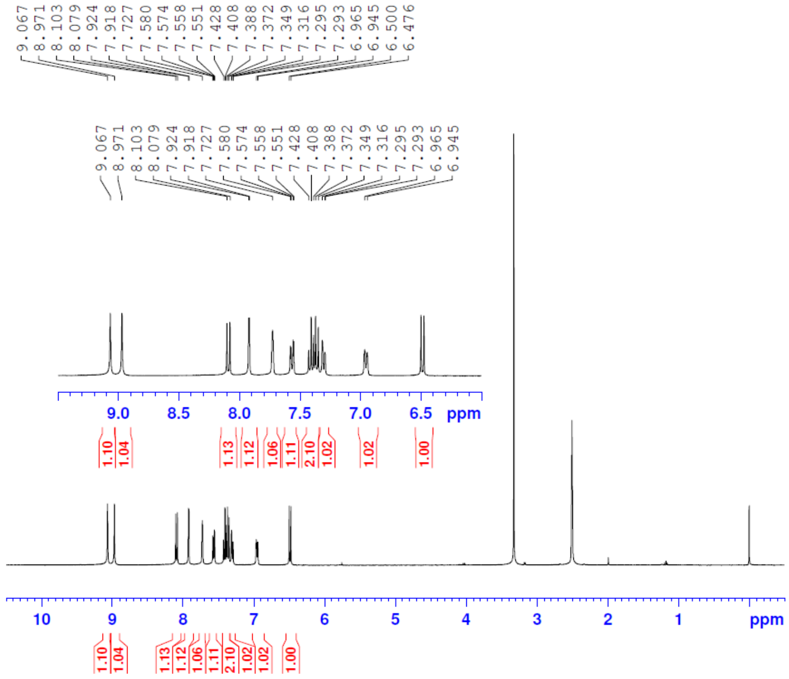

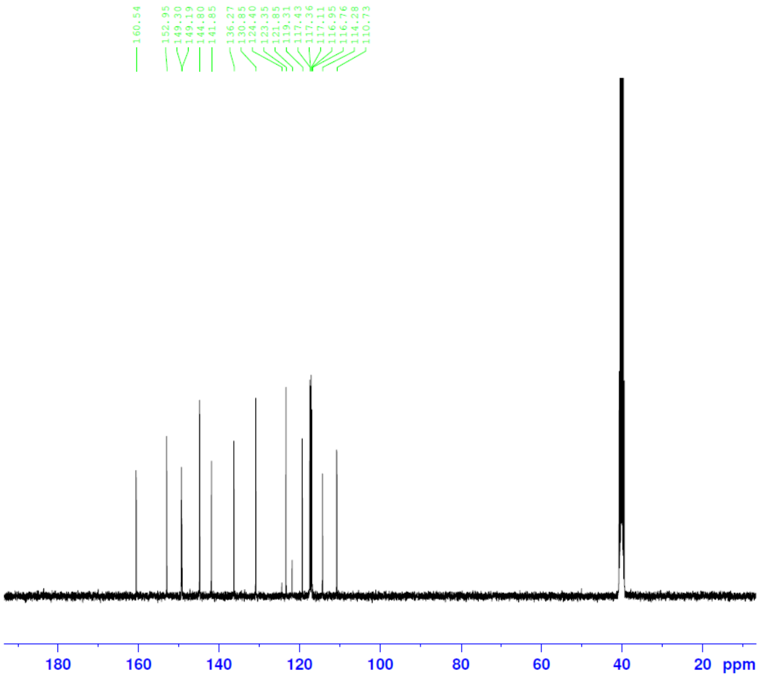

2) HRMS chart of 3q

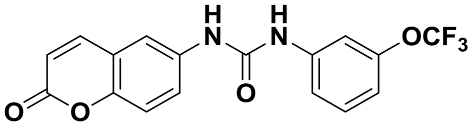

3q

HRMS (EI) m/z calcd. for $C_{17}H_{12}F_3N_2O_4$ $[M+H]^+$: 365.0749, found 365.0742

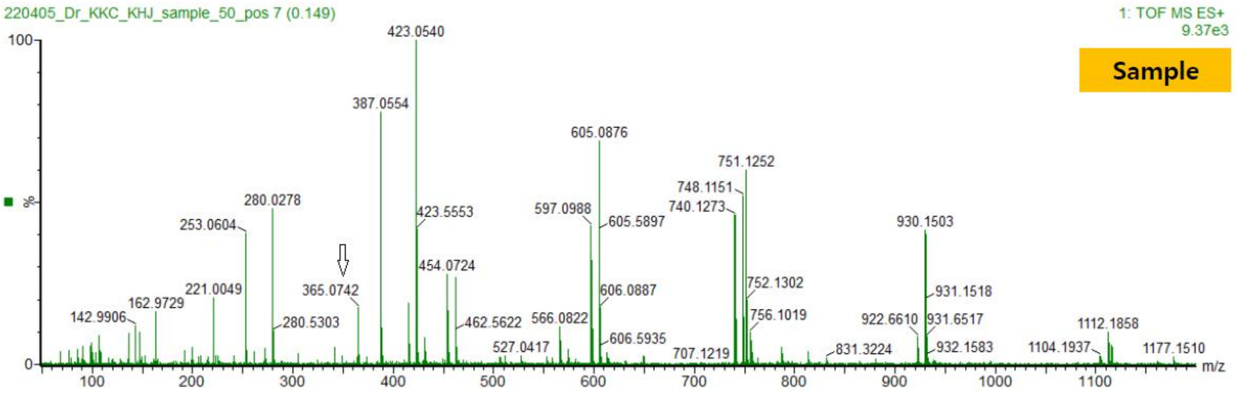

3) Molecular dynamics simulations

S1. MD simulations

The molecular dynamics (MD) simulations were conducted utilizing the Desmond simulation software developed by Schrödinger LLC [1]. The NPT ensemble was utilized in all experimental runs, with a temperature of 300 K and a pressure of 1 bar. The duration of the simulation was 200 nanoseconds, during which the ligands underwent relaxation at a time scale of 1 picosecond. All simulations utilized the OPLS3 force field parameters [2]. The cutoff radius utilized in Coulomb interactions was determined to be 9.0 Å. The limits of the orthorhombic periodic box were established at a distance of 10 Å from the atoms of the protein. The water molecules were explicitly characterized utilizing the transferable intermolecular potential with three points (TIP3P) model [3, 4]. Salt concentration set to 0.15 M NaCl and was built using the System Builder utility of Desmond [5]. The Martyna−Tuckerman−Klein chain coupling scheme with a coupling constant of 2.0 ps was used for the pressure control and the Nosé−Hoover chain coupling scheme for the temperature control [6, 7]. The calculation of nonbonded forces was performed using a RESPA integrator. In this approach, the short-range forces were updated at every step, while the long-range forces were updated every three steps. The trajectories were recorded at intervals of 20 nanoseconds for the purpose of analysis. The analysis of the behavior and interactions between the ligands and protein was conducted using the Simulation Interaction Diagram tool, which is a component of the Desmond MD package. The stability of molecular dynamics (MD) simulations was assessed by monitoring the root mean square deviation (RMSD) of the ligand and protein atom positions over time.

S2. MD trajectory analysis and prime MM-GBSA calculations

The Maestro software's simulation interactions diagram panel was utilized to monitor the contribution of interactions in the stability of the ligand-protein complex. The ligand binding free energies and ligand strain energies for docked compounds were calculated using the molecular mechanics generalized born/solvent accessibility (MM-GBSA) method. This calculation was performed over a duration of the last 50 ns using the thermal_mmgbsa.py python script provided by Schrodinger. The script takes a Desmond trajectory file, divides it into separate snapshots, conducts MM-GBSA calculations on each frame, and generates the average computed binding energy as the output.

References

1. Release, S., *3: Desmond molecular dynamics system, DE Shaw research, New York, NY, 2017.* Maestro-Desmond Interoperability Tools, Schrödinger, New York, NY, 2017.
2. Harder, E., et al., *OPLS3: a force field providing broad coverage of drug-like small molecules and proteins.* Journal of chemical theory and computation, 2016. **12**(1): p. 281-296.
3. Jorgensen, W.L., et al., *Comparison of simple potential functions for simulating liquid water.* The Journal of chemical physics, 1983. **79**(2): p. 926-935.
4. Neria, E., S. Fischer, and M. Karplus, *Simulation of activation free energies in molecular systems.* The Journal of chemical physics, 1996. **105**(5): p. 1902-1921.
5. Manual, D.U., *Desmond2. 2.* 2009.
6. Martyna, G.J., M.L. Klein, and M. Tuckerman, *Nosé–Hoover chains: The canonical ensemble via continuous dynamics.* The Journal of chemical physics, 1992. **97**(4): p. 2635-2643.
7. Martyna, G.J., D.J. Tobias, and M.L. Klein, *Constant pressure molecular dynamics algorithms.* The Journal of chemical physics, 1994. **101**(5): p. 4177-4189.

4) DDR1/2 inhibition curves

IC50 curves for DDR1	IC50 curves for DDR2

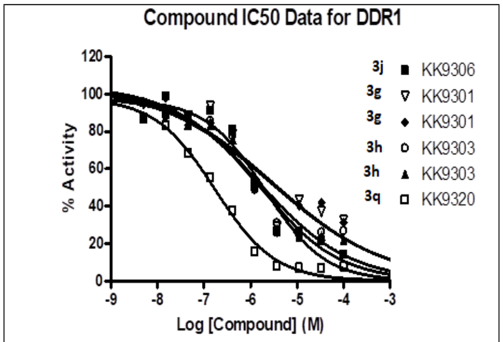

	3i KK9306	3g KK9301	3g KK9301	3h KK9303	3h KK9303	3q KK9320
HILLSLOPE	-0.6789	-0.3612	-0.3584	-0.4518	-0.5208	-0.6753
EC50	2.067e-006	2.980e-006	2.826e-006	1.574e-006	1.737e-006	1.732e-007

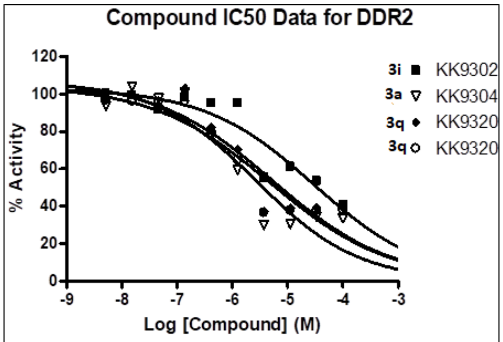

	3i KK9302	3a KK9304	3q KK9320	3q KK9320
HILLSLOPE	-0.4393	-0.4712	-0.4045	-0.4033
EC50	2.994e-005	2.729e-006	5.263e-006	4.901e-006

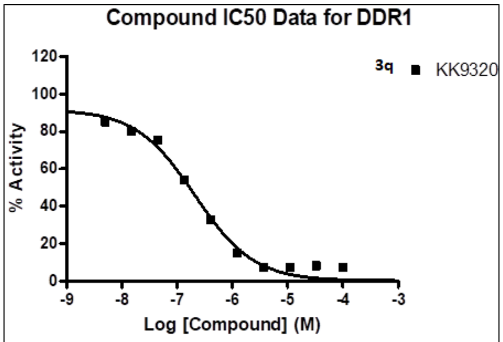

	3q KK9320
HILLSLOPE	-0.8146
EC50	2.082e-007

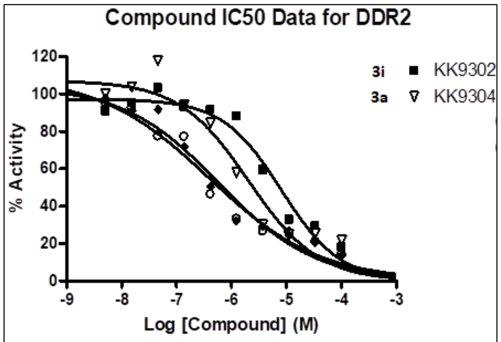

	KK9302	KK9304
HILLSLOPE	-0.8084	-0.6854
EC50	8.042e-006	2.041e-006

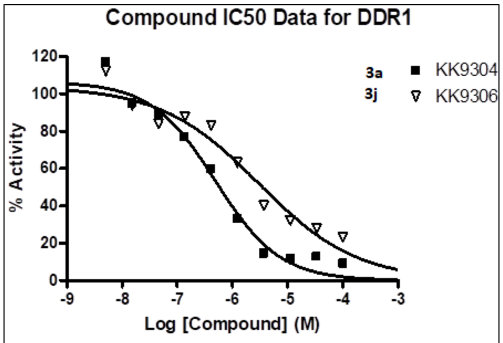

	3a KK9304	3j KK9306
HILLSLOPE	-0.7471	-0.4872
EC50	4.943e-007	2.975e-006

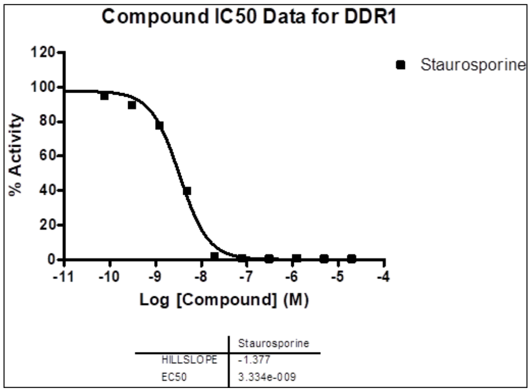
Compound IC50 Data for DDR1
% Activity
Log [Compound] (M)
Staurosporine
Staurosporine
HILLSLOPE -1.377
EC50 3.334e-009

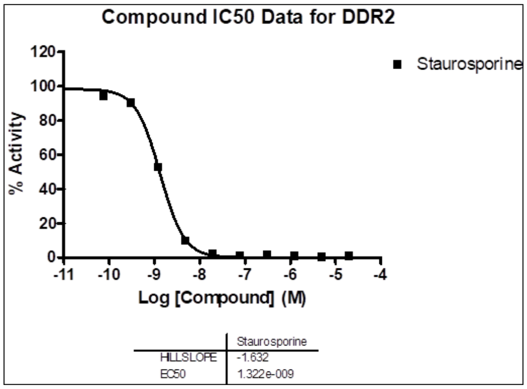
Compound IC50 Data for DDR2
% Activity
Log [Compound] (M)
Staurosporine
Staurosporine
HILLSLOPE -1.632
EC50 1.322e-009

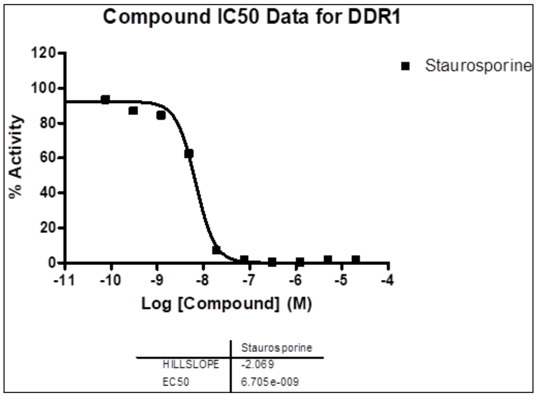
Compound IC50 Data for DDR1
% Activity
Log [Compound] (M)
Staurosporine
Staurosporine
HILLSLOPE -2.069
EC50 6.705e-009

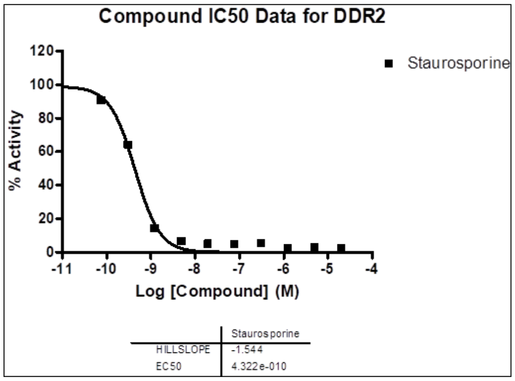
Compound IC50 Data for DDR2
% Activity
Log [Compound] (M)
Staurosporine
Staurosporine
HILLSLOPE -1.544
EC50 4.322e-010

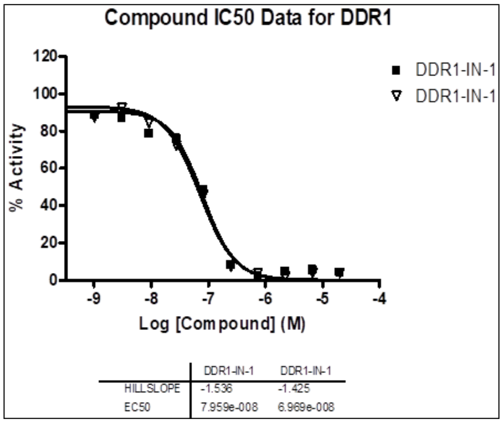
Compound IC50 Data for DDR1
% Activity
Log [Compound] (M)
DDR1-IN-1
DDR1-IN-1
DDR1-IN-1 DDR1-IN-1
HILLSLOPE -1.536 -1.425
EC50 7.959e-008 6.969e-008

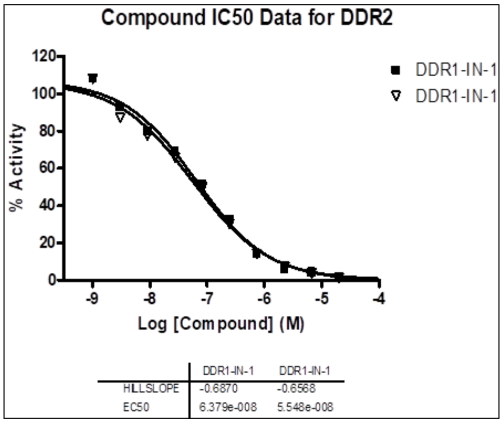
Compound IC50 Data for DDR2
% Activity
Log [Compound] (M)
DDR1-IN-1
DDR1-IN-1
DDR1-IN-1 DDR1-IN-1
HILLSLOPE -0.6870 -0.6568
EC50 6.379e-008 5.548e-008

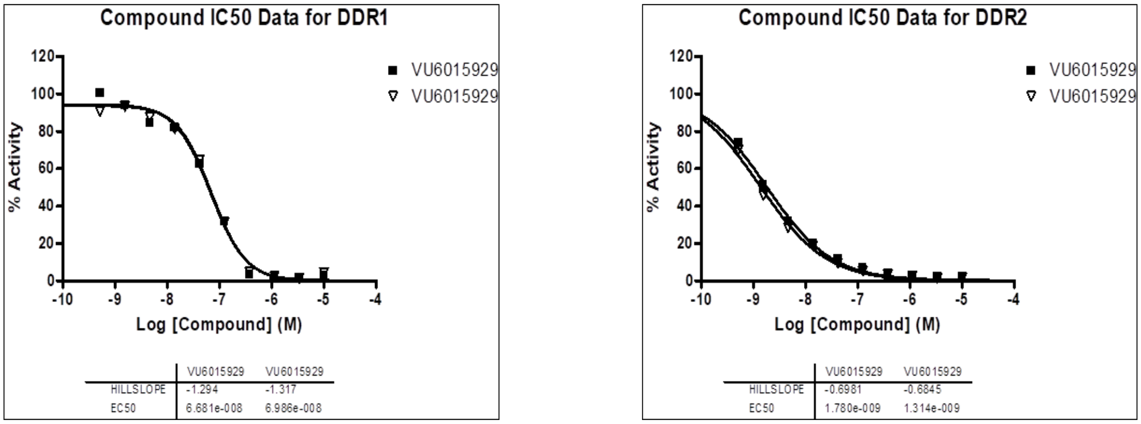

Figure S1. IC$_{50}$ curves of compounds **3a**, **3g–j**, **3q**, **Staurosporine**, **DDR1-In-1**, and **VU6015929** with DDR1 (left panel) and DDR1 (right panel).

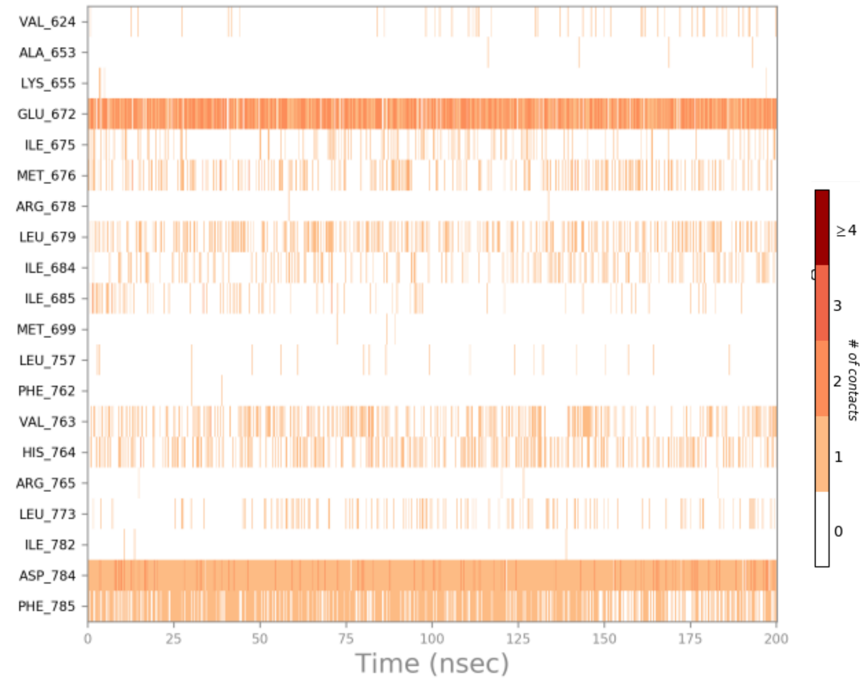

(a)

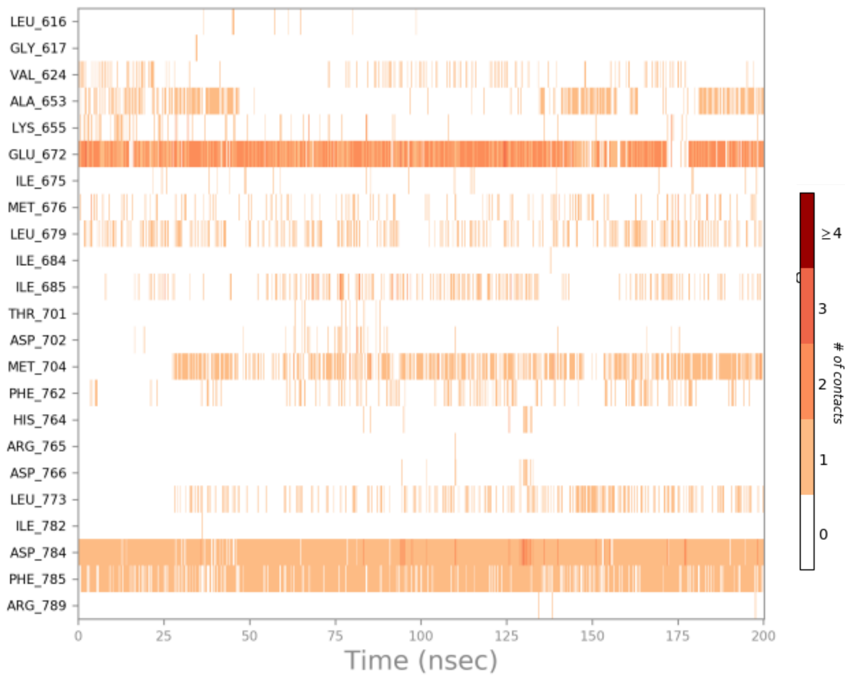
LEU_616
GLY_617
VAL_624
ALA_653
LYS_655
GLU_672
ILE_675
MET_676
LEU_679
ILE_684
ILE_685
THR_701
ASP_702
MET_704
PHE_762
HIS_764
ARG_765
ASP_766
LEU_773
ILE_782
ASP_784
PHE_785
ARG_789
0
25
50
75
100
125
150
175
200
Time (nsec)
≥4
3
2
1
0
# of contacts

(b)

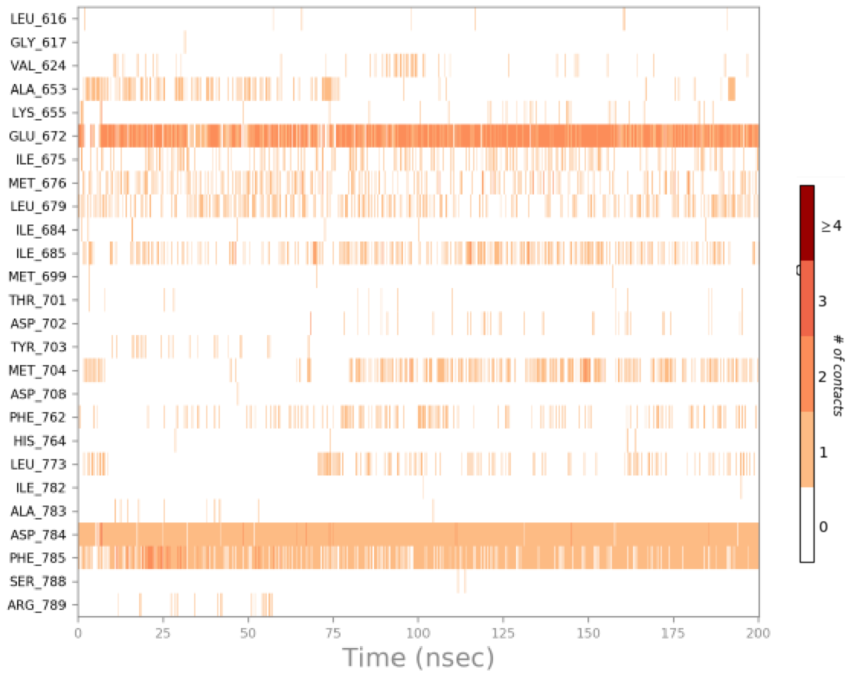
LEU_616
GLY_617
VAL_624
ALA_653
LYS_655
GLU_672
ILE_675
MET_676
LEU_679
ILE_684
ILE_685
MET_699
THR_701
ASP_702
TYR_703
MET_704
ASP_708
PHE_762
HIS_764
LEU_773
ILE_782
ALA_783
ASP_784
PHE_785
SER_788
ARG_789
0
25
50
75
100
125
150
175
200
Time (nsec)
≥4
3
2
1
0
# of contacts

(c)

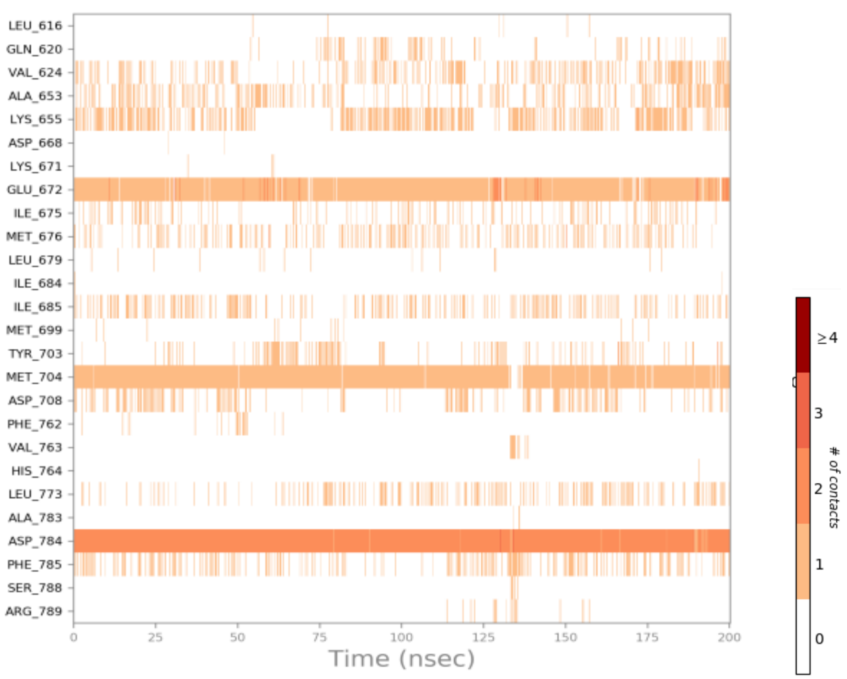

(d)

Figure S2. Heat map showing the total number of DDR1 protein (PDB ID: 5FDP)-ligand interactions all over the simulation time of 200 ns for (**a**) **3a**, (**b**) **3i**, (**c**) **3q**, and (**d**) **Co**.
